# Mycophenolate mofetil for autoimmune cytopenias in children: high rates of response in inborn errors of immunity

**DOI:** 10.3389/fped.2023.1174671

**Published:** 2023-10-17

**Authors:** Rubén Berrueco, Elisa González-Forster, Angela Deya-Martinez, María Solsona, Ana García-García, Joan Calzada-Hernández, Luo Yiyi, Alexandru Vlagea, Anna Ruiz-Llobet, Laia Alsina

**Affiliations:** ^1^ Pediatric Hematology Department, Hospital Sant Joan de Déu, Barcelona, Spain; ^2^ Institut de Recerca Sant Joan de Déu de Barcelona (IRSJD), Barcelona, Spain; ^3^ Instituto Nacional de Investigación Biomédica en Enfermedades Raras (CIBER ER), Instituto de Salud Carlos III, Madrid, Spain; ^4^ Clinical Immunology and Primary Immunodeficiencies Unit, Pediatric Allergy and Clinical Immunology Department, Hospital Sant Joan de Déu, Barcelona, Spain; ^5^ Clinical Immunology Unit, Hospital Sant Joan de Déu-Hospital Clínic Barcelona, Barcelona, Spain; ^6^ Pediatric Rheumatology Unit, Hospital Sant Joan de Déu, Universitat de Barcelona, Barcelona, Spain; ^7^ Immunology Department, Centre of Biomedical Diagnosis, Hospital Clínic, Barcelona, Spain; ^8^ Department of Surgery and Surgical Specializations, Facultat de Medicina I Ciències de la Salut, Universitat de Barcelona, Barcelona, Spain

**Keywords:** pediatrics, autoimmune hemolytic anemia, autoimmune thrombocytopenia, Evans syndrome, autoimmune diseases, mycophenolate mofetil

## Abstract

Second-line treatments of autoimmune cytopenias (AC) are not well-defined in children. Mycophenolate mofetil (MMF) is an immunosuppressant agent that has been demonstrated to be safe and effective in this setting. A retrospective observational study was conducted in 18 children with prolonged AC who received MMF, in order to describe clinical and biological markers of response. The overall response rate of MMF at 20–30 mg/kg per day was 73.3%. All patients with Evans syndrome (*n* = 9) achieved complete response. Among the patients with monolineage AC (*n* = 9), those with an underlying inborn errors of immunity (IEI), tended to respond better to MMF. No biological markers related to treatment response were found. Rather, lymphocyte subpopulations proved useful for patient selection as a marker suggestive of IEI along with immunoglobulin-level determination.

## Introduction

Autoimmune cytopenias (AC) are characterized by immune-mediated destruction of one or more hematopoietic lineage cells. When treatment is needed in pediatric patients with immune thrombocytopenia (ITP), first-line options are well-defined (1, 2). However, lack of studies in chronic ITP and other disorders such as autoimmune hemolytic anemia (AIHA), autoimmune neutropenia (AIN), and Evans syndrome (ES) during infancy makes it difficult to tailor second-line treatments (3).

Mycophenolate mofetil (MMF) is an immunosuppressant agent that reduces T- and B-cell proliferation by inhibiting inosine monophosphate dehydrogenase without a relevant infection risk (4, 5). This drug has been demonstrated to be safe and effective for the treatment of AC in children, and it is particularly effective in patients with underlying ES or autoimmune lymphoproliferative syndrome (ALPS), with variable response rates of 65% up to 92% (4–7). It has been proposed that its mechanism of action is rebalancing of the underlying T-cell dysregulation. Nevertheless, clear biological makers to predict MMF response are lacking (5, 8).

The aim of the present study is to analyze the outcome of a cohort of pediatric patients with AC who received MMF as second- or further-line treatment and to evaluate possible clinical and biological markers predictive of response to treatment.

## Methods

We present a retrospective observational study that includes children below the age of 18 years diagnosed with AC and treated with MMF in a tertiary university pediatric hospital between January 2009 and January 2022. The MMF treatment was initiated at 20 mg/kg per day BID. The need for special attention to possible gastrointestinal toxicity was explained to the patients and caregivers. A dose increase of up to 30 mg/kg per day BID was indicated in those patients who did not achieve a partial or complete response (CR) after 2–4 weeks of treatment (4, 6, 7). A minimum of 3 months of MMF was required to include the patient in the study.

**Figure 1:**
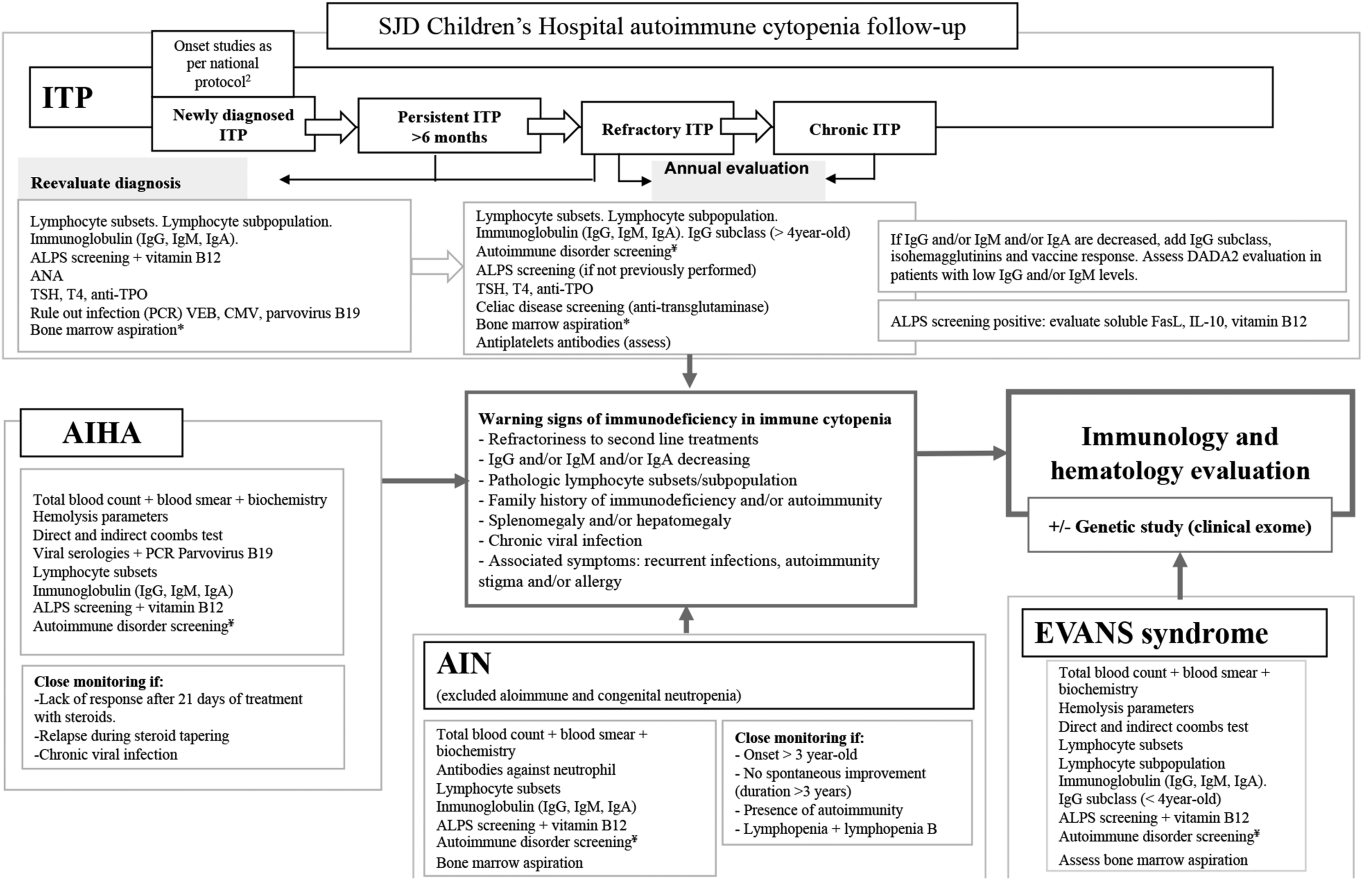
SJD Children's Hospital autoimmune cytopenia follow-up. SJD, Sant Joan de Deú Hospital; ITP, immune thrombocytopenia; ALPS, autoimmune lymphoproliferative syndrome; TSH, thyrotropin; TPO, thyroid peroxidase; AIHA, autoimmune hemolytic anemia. *If it has not been previously performed. ¥ Autoimmune disorder screening: ANA, anti-dsDNA, anti-ENA, antiphospholipid syndrome screening including lupus anticoagulant, anticardiolipin, anti-beta-2-glycoprotein I; C3, C4, CH50, urine basis.

A protocol implemented in our hospital for the evaluation of an underlying pathology in chronic persistent AC is mainly oriented at ruling out inbor errors of immunity (IEI), rheumatologic/autoimmune diseases such as systemic lupus erythematosus (SLE), and other connective tissue diseases, endocrine–metabolic disorders, and chronic infection. It includes (Figure 1) (5, 8, 9) a complete immune evaluation with lymphocyte T/B/NK and T/B subpopulations, immunoglobulin (IgG, IgM, IgA) and IgG subclasses levels (ARCHITECT c Systems and ASEROSET System, immunoturbidimetric measure), vaccine responses, and autoantibodies. Specifically, the B-cell compartment includes LB naïve (IgM + IgD + CD27-), LB memory (IgD-), activated LB (CD21^low^ CD38^low^), and Bregs (CD24^hi^CD38^hi^). The T-cell compartment includes CD3 + TcR*γδ*, CD4 naïve (CD4 + CCR7 + CD45RA+), CD8 naïve (CD8 + CCR7 + CD45RA+), and the expression of HLA-DR in both CD4+ and CD8+ LT (flow cytometry using BD Biosciences, San Jose, CA, USA, FACS Canto II), ALPS screening (flow cytometry for CD4/CD8 double negative, alpha–beta+, vitamin B12), and genetic test (gene panel, Agilent Technologies, v5.3.0, which includes 400 genes linked to IEI, of the 485 genes in the updated 2022 IUIS classification) (10). Subsequent lymphocyte subpopulation evaluation of patients is annually performed to identify a developing IEI.

Medical charts were reviewed to retrieve data such as gender, age, date of diagnosis, AC etiology, previous treatment, indication for MMF, dose, date of response, duration of treatment and relapse, and the above-mentioned immunological and genetic variables. The response to treatment was considered according to the international criteria for patients with ITP (11). In patients with AIN, a CR was defined as the maintained level of neutrophils without G-CSF treatment at >1.5 × 10^9^/L, partial response (PR) as neutrophils without G-CSF treatment at 0.5–1.5 × 10^9^/L, and no response (NR) when neutrophils were <0.5 × 10^9^/L instead of treatment. In patients with AIHA, CR was defined as the maintained hemoglobin level without red blood cell transfusion at >11.5 g/dl, PR as the maintained hemoglobin level without red blood cell transfusion at 8.5–11.5 g/dl, and NR as the hemoglobin level at <8.5 g/dl or transfusion dependent.

When present, data regarding lymphocyte subpopulation before and during the MMF treatment were also collected. An IEI was diagnosed if the patient fulfilled the diagnostic criteria defined by the European Society for Immunodeficiencies and/or a confirmed pathogenetic mutation in a known IEI gene was identified (10, 12). The Sant Joan de Deu Ethics Committee approved this study (EOM-05-22). The data that support the findings of this study are available from the corresponding author upon reasonable request.

## Results

### Cohort description

During the study period, a total of 475 patients were diagnosed with some type of AC: ITP = 364, AHAI = 43, AIN = 51, ES = 15, and ALPS = 2. Of these, 18 patients (4.2%) received MMF during their follow-up (Table 1). Nine patients had an ES: five related to IEI, one to infection still not fulfilling IEI diagnostic criteria, and three idiopathic. Their mean age was 12.4 ± 8.5 years, and 55% were girls. Among them, three patients received MMF as the first-line treatment. Another nine patients had an isolated AC: seven had been diagnosed with ITP (two related to rheumatic diseases and two to IEI; their mean age was 8.7 ± 4.4 years, and 57% were girls), and two patients had an AIHA (one related to infection and one to IEI). None of the patients with AC received MMF as the first-line treatment.

**Table 1:** Main patient characteristics including autoimmune cytopenia etiology, previous treatment, and response to MMF.

Patient		Age	Gender	Etiology	Previous treatments				MMF	Time to CR (months)	Time to withdrawal (months) after NR	Follow-up
					Steroids	IVIG	ar-TPO	Others				
Evans syndrome												
1	ITP + AIHA + AIN	14.2	F	Infection (herpes zoster)	TR				CR	2.2	—	
2	ITP + AIHA	7.8	F	Idiopathic	NR	NR	NR (Rm/El)		CR	6.5	—	
3	ITP + AIN	12.5	M	ID: Kabuki syndrome	NR	NR			CR (+El)^b^	0.7	—	
4	ITP + AIN	13.5	M	Idiopathic	PR			NR (AZA)	CR	4.7	—	
5	ITP + DCT	15.9	M	CVID					CR	1.0	—	
6	ITP + AIN	9.2	F	CVID^a^	PR	PR			CR	2.4	—	
7	ITP + AIHA + AIN	12.8	F	CVID^a^					CR	0.4	—	
8	ITP + AIN	11.0	M	ID: 22q11 deletion	PR	TR			CR (+El)^b^	1.0	—	
9	ITP + AIN + DCT	14.9	F	Idiopathic					CR	0.6	—	
Autoimmune cytopenia												
10	ITP	12.7	F	MCTD^a^	PR	NR	PR (Rm)		NR		5.1	CR after HXC + El
11	ITP	4.5	M	Idiopathic	NR	PR	NR		NR		3.0	CR (splenectomy)
12	ITP	9.2	F	SLE^a^	PR	PR		NR (HXC)	NR		6.1	CR: rituximab
13	ITP	2.1	M	Idiopathic	NR	PR	NR (Rm/El)		NR		3.4	NR
14	ITP	13.3	F	CVID	PR	PR-TR	NR (Rm/El)		CR (+Rm)^b^	1.2	—	
15	ITP	6.7	M	Idiopathic	NR	NR	NR (Rm/El)		NR		7.8	CR: rituximab
16	ITP	12.6	F	CVID (IKZF1 mutation)	PR				Initial PR CR (+sIVIG)^c^	27.1	—	
17	AIHA	11.7	M	Infection: Parvovirus B19		PR			CR	0.5	—	
18	AIHA	3.8	M	ALPS (CASP10 mutation)	PR				Initial PR CR (+sIVIG)^c^	14.3	—	

ITP, immune thrombocytopenia; AIHA, autoimmune hemolytic anemia; AIN, autoimmune neutropenia; DCT, direct Coombs test positive without hemolysis; M, masculine; F, feminine; CVID, common variable immunodeficiency; ID, immunodeficiency; MCTD, mixed connective tissue disease; SLE, systemic lupus erythematosus; IVIG, intravenous immunoglobulin (treatment dose: 0.8–1 g/kg/dose); sIVIG, intravenous immunoglobulin (substitutive dose: 0.5 g/kg/dose); ar-TPO, thrombopoietin analogs; Rm, romiplostim; El, eltrombopag; AZA, azathioprine; HXC, hydroxychloroquine; CR, complete response; PR, partial response; NR, no response; TR, transient response. In patients with AIN, CR, maintained level of neutrophils without G-CSF treatment at >1.5 × 10^9^/L; PR, neutrophils without G-CSF treatment at 0.5–1.5 × 10^9^/l; and NR, neutrophils at <0.5 × 10^9^/l. In patients with AIHA, CR, maintained hemoglobin level without red blood cell transfusion at >11.5 g/dl; PR, maintained hemoglobin level without red blood cell transfusion at 8.5–11.5 g/dl; and NR, hemoglobin level at <8.5 g/dl or transfusion dependent.

^a^ Patients diagnosed during follow-up.

^b^ MMF and ar-TPO were administered together. After achieving a CR, eltrombopag and romiplostim were withdrawn (no relapse).

^c^ Bitherapy with substitutive IVIG (treatment ongoing in both cases).

To sum up, an underlying disorder triggering persistent AC was identified in 12 out of 18 patients.

### Mycophenolate mofetil treatment course

After a median time of 2.2 months (range 0.4–27.1) of treatment with MMF at 20–30 mg/kg per day, 72.3% (*n* = 13/18) of the patients achieved a complete response (100% of ES patients). Two patients with isolated AC and underlying IEI initially had a partial response but finally achieved a complete remission after 14 and 27 months of treatment. None of the them developed relevant secondary side effects to MMF treatment. The patients who were considered non-responders had received MMF for a range of 2.5–3 months before its withdrawal.

After completing a 2-year treatment period, MMF was gradually withdrawn in three patients (patients 1, 3, and 4 in Table 1). Among them, one patient with ES (patient 1) relapsed 6 months after complete withdrawal. Treatment with MMF was restarted at 20 mg/kg, and a new complete response was achieved 2 months after reinitiating.

### Immune evaluation

As per the protocol, a complete immune evaluation was performed in all patients. This led to the diagnosis of an underlying immune-mediated disorder triggering persistent AC in nine out of 18 patients (two with rheumatic diseases and seven with IEI) (Table 1).

Subsequent yearly lymphocyte subpopulation evaluation during the MMF treatment was performed in 10 out of 18 patients (Table 2). None of them showed relevant modifications in their immune profile associated with MMF administration during follow-up. However, periodic immune evaluation revealed an underlying condition (IEI) in four patients (Table 1). Only one patient (patient 6) showed high LB CD21^low^ in the lymphocyte population at baseline. She had a complete response to MMF, but levels of LB CD21^low^ remain without changes to date.

**Table 2:** Lymphocyte population at baseline.

Patient	Lymphocyte B compartment			Lymphocyte T compartment				
	LB naive (IgM + IgD + CD27-)	LB memory (IgD-)	CD21low CD38 low	CD3+ TcRγδ	CD4 naïve (CCR7 + CD45RA+)	CD8 naïve (CCR7 + CD45RA+)	HLA-DR expression in LT CD4+	HLA-DR expression in LT CD8+
1	89% (*N*)	10 (*N*)	6.2% (*N*)	3% (low)	46.6% (*N*)	49.6%	6.2% (*N*)	10.9% (*N*)
2	85.6% (*N*)	14.4% (*N*)	5.5% (*N*)	16.3% (high)	68.6% (high)	71.3% (high)	2.9% (low)	5.2% (*N*)
3	94.7% (*N*)	5.2% (*N*)	6.9% (*N*)	11.7% (*N*)	13.1% (low)	5.3% (low)	33% (high)	70.4% (high)
6	91.8% (*N*)	8.2% (*N*)	22.3% (high)	3.9% (*N*)	31.3% (*N*)	53.7% (*N*)	19.2% (high)	15.8% (*N*)
7	89.8% (*N*)	10.3% (*N*)	2.1% (*N*)	6.7% (*N*)	47.9% (*N*)	57.1% (*N*)	2.7% (low)	3.7% (low)
9	91.8% (*N*)	8.2% (*N*)	1.1% (*N*)	8.9% (*N*)	55.9% (*N*)	55.4% (*N*)	1.9% (low)	1.3% (low)
14	92.7% (*N*)	7.3% (*N*)	1.5% (*N*)	5% (*N*)	57.8% (*N*)	59% (*N*)	2.8% (low)	12.6% (*N*)
15	93.3% (*N*)	6.7% (*N*)	2.9% (*N*)	4.8% (*N*)	57.9% (*N*)	64.8% (high)	4.4% (*N*)	11.2% (*N*)
16	91% (*N*)	9% (*N*)	2.3% (*N*)	5.7% (*N*)	59.2 (high)	65.7% (high)	2.5% (low)	6.5% (*N*)
17	94.3% (*N*)	5.7% (*N*)	2.5% (*N*)	3.8% (*N*)	36.8% (*N*)	52.7% (*N*)	13.6% (high)	10% (*N*)

## Discussion

MMF has previously been demonstrated to be safe and effective for the treatment of AC in children (4, 5, 7). Our study reveals a high response rate to MMF in a series of carefully selected pediatric patients with persistent AC (72.3%, 13 out of 18 patients), of whom nine responders had an underlying immune-mediated disease. Nevertheless, it was not possible to identify any biological maker predictive of a good response to MMF beyond the alterations themselves associated with the diagnosis of IEI.

The presence of an underlying immune-mediated disease in a cohort of pediatric patients presenting with chronic persistent AC is not surprising. Indeed, as much as 25% of patients with IEI presented with autoimmune symptoms as initial manifestation of the IEI between ages 6 and 25 years (13), with AC being the most common autoimmune manifestation, AIHA in particular (14). Moreover, in children with ES, an underlying IEI or related gene mutation can be identified in as much as 50% of the patients (15, 16). Finally, in a cohort of patients with pediatric common variable immunodeficiency (CVID), symptoms of immune dysregulation were frequent (82%), and AC was present in 46.5% of the evaluated patients (17). In fact immune alterations observed in six patients with pediatric CVID included in this cohort met the working definitions of the European Society for Immunodeficiencies (ESID) Registry for the clinical diagnosis of CVID, consisting of at least one of the following: (1) increased susceptibility to infection, (2) autoimmune manifestations, (3) granulomatous disease, (4) unexplained polyclonal lymphoproliferation, and (5) affected family member with antibody deficiency and marked decrease of IgG and marked decrease of IgA with or without low IgM levels (measured at least twice; <2 SD of the normal levels for their age) and at least one of the following: (1) poor antibody response to vaccines (and/or absent isohemagglutinins), i.e., absence of protective levels despite vaccination where defined, and (2) low-switched memory B-cells (<70% of age-related normal value) and secondary causes of hypogammaglobulinemia ruled out (e.g., infection, protein loss, medication, malignancy) (12).

According to the above results (6, 7), patients with ES had a better response to MMF than those with monolineage AC. This could be related to an underlying immunological disorder such as IEI that may benefit from MMF as immunosuppressive treatment to rebalance the underlying T-cell dysregulation (3, 8). In fact, among the patients with isolated AC, only those with CVID responded to MMF, which reinforces the idea that IEI patients are the likeliest to benefit from MMF as suggested recently (3).

Regarding other clinical predictors, it has been suggested that a prompt initiation of MMF could be associated with better responses (5, 18). In this regard, six out of nine patients with ES achieved a quick response during the first month of treatment, including three patients who received MMF as the first-line treatment. In contrast, patients with isolated AC who started MMF as a third- or fourth-line treatment barely responded to treatment. This was surprising, as a previously published short series in pediatric patients with ITP like ours showed an excellent response rate (5). It may be that they were administered a concomitant treatment with corticosteroids, which may possibly have contributed to this response rate. We tend to avoid steroid co-administration, but we associated thrombopoietin analogs to MMF in three patients with good outcome. However, the number of patients is too low to draw any conclusion. Thus, the impact of early (first-line treatment) as opposed to late MMF initiation (third- or fourth-line treatment) is difficult to evaluate since the time from diagnosis to MMF initiation was variable mainly depending on the type of AC (ES as first-line vs. other AC, later on in the course of the disease based on the underlying disease and the existence of previous partial response to treatment).

Since the role of MMF in AC relies on its capacity to regulate T-cell dysfunction, we evaluated lymphocyte subpopulations before and during the treatment with MMF, since reduced isotype-switched memory B-cells (≤0.55% of B-cells), increased CD21^low^ B-cells (>10%), and reduced T-cells (CD4) have been linked to an increased risk of non-infectious complications in CVID (19, 20). The latter was only possible in 10 patients, which is a limitation in itself, but we did not find any relevant trend in lymphocyte subpopulation changes through treatment. It is possible that prospective studies evaluating further clinical and biological predictors of good response to MMF will be able to draw conclusions in this regard. In addition, recently described biomarkers linked to immune dysregulation in IEI, and in CVID in particular, such as T follicular helper (Tfh), were not included in the study. Indeed, Tfh assists the activation, proliferation, and differentiation of B-cells into plasma cells and thus regulate host antibody response (21–23). In contrast, Breg cells, a newly designated B-cell subset group, appear to prevent T-cell differentiation. Tfh and Breg are linked since Tfh cells secrete IL-21 and thus facilitate Breg cell differentiation. Thus, Tfh and Breg cells have been reported to play essential roles in humoral immunity, especially in inflammation and autoimmune diseases (24). Nevertheless, no specific pattern of Breg cells (systematically increased or decreased) was observed in our cohort.

Based on our experience, extended immune evaluation to better select the patients with plausible immune dysregulation (associated with rheumatic diseases or IEI) is related to the high rate of response observed in our series. Accordingly, and considering the limitations of the retrospective data, we suggest that MMF be used early on in the course of a persistent AC, even as a first- or second-line treatment in those patients with ES and/or those patients with AC refractory to other treatments (3), especially in those cases in which CVID is suspected or previously diagnosed. In this regard, we recommend performing periodic evaluations (including genetic testing) so as to rule out the underlying IEI or other disorders, which were identified in as many as 65% of patients, as recently reported in a large ES cohort.

## Data Availability

The original contributions presented in the study are included in the article/Supplementary Material, further inquiries can be directed to the corresponding author.
